# Bax Inhibitor-1, a Conserved Cell Death Suppressor, Is a Key Molecular Switch Downstream from a Variety of Biotic and Abiotic Stress Signals in Plants

**DOI:** 10.3390/ijms10073149

**Published:** 2009-07-10

**Authors:** Naohide Watanabe, Eric Lam

**Affiliations:** Biotechnology Center for Agriculture and the Environment, Rutgers University, 59 Dudley Road, New Brunswick, NJ 08901, USA; E-Mail: watanabe@aesop.rutgers.edu (N.W.)

**Keywords:** abiotic stress, Bax Inhibitor-1, biotic stress, ER stress, programmed cell death

## Abstract

In Nature plants are constantly challenged by a variety of environmental stresses that could lead to disruptions in cellular homeostasis. Programmed cell death (PCD) is a fundamental cellular process that is often associated with defense responses to pathogens, during development and in response to abiotic stresses in fungi, animals and plants. Although there are many characteristics shared between different types of PCD events, it remains unknown whether a common mechanism drives various types of PCD in eukaryotes. One candidate regulator for such a mechanism is Bax Inhibitor-1 (BI-1), an evolutionary conserved, endoplasmic reticulum (ER)-resident protein that represents an ancient cell death regulator that potentially regulates PCD in all eukaryotes. Recent findings strongly suggested that BI-1 plays an important role in the conserved ER stress response pathway to modulate cell death induction in response to multiple types of cell death signals. As ER stress signaling pathways has been suggested to play important roles not only in the control of ER homeostasis but also in other biological processes such as the response to pathogens and abiotic stress in plants, BI-1 might function to control the convergence point that modulates the level of the “pro-survival and pro-death” signals under multiple stress conditions.

## 1. Introduction

Programmed cell death (PCD) comprises a series of genetically controlled cell suicide processes and plays a fundamental role in various biological processes including cell proliferation, generation of developmental patterns, defenses of eukaryotes against pathogens and environmental insults [1–3]. One of the most widely studied forms of PCD is apoptosis, a type of PCD that displays distinct physiological and morphological features in animal cells. Elucidation of the molecular architecture of metazoan PCD have unraveled the immense diversity of signal transduction pathways and the enormous multitude of components for the cell death engine [1,4,5]. Some of the regulatory networks were discovered by the use of model organisms such as *Drosophila melanogaster* and *Caenorhabditis elegans* [4–6]. However, many aspects for the precise control mechanisms of PCD and the sequence of biochemical events that ultimately leads to cellular suicide remain to be elucidated.

The genes that control apoptosis can be conserved across wide evolutionary distances in metazoans [4–6]. For example, metazoan apoptosis is predominantly controlled through Bcl-2-related proteins [7–9]. So far 15 mammalian family members were identified, which were divided into three subfamilies: Bcl-2 subfamily (pro-survival type; Bcl-2, Bcl-X_L_, Bcl-w, Mcl-1 and A1), Bax subfamily (pro-apoptotic type; Bax, Bak and Bok) and BH3 subfamily (pro-apoptotic type; Bad, Bid, Bik, Blk, Hrk, BNIP3 and BimL). The Bcl-2 subfamily proteins inhibit apoptosis, whereas Bax subfamily proteins activate apoptosis. Bax and Bak actively induce cytochrome *c* release from mitochondria within cells, which is inhibited by anti-apoptotic Bcl-2 family members. BH3 subfamily proteins share a short motif with Bcl-2 family proteins and regulate their activity antagonistically through their direct interaction. One model posits that upon apoptosis initiation, BH3-only proteins are induced and then bind and inhibit anti-apoptotic Bcl-2 family proteins, allowing pro-apoptotic Bax and Bak to permeabilize the mitochondrial outer membrane releasing cytochrome c and other proteins to activate caspases and eventually induce cell death [7–9].

Under certain conditions, some biotic or abiotic stresses such as pathogen attack, heat shock, ultraviolet (UV) C irradiation, ozone exposure, phytotoxin, or oxidative stress-inducing agents could cause a number of structural and biochemical changes characteristics of apoptosis in plant cells [10–12]. These cytological and biochemical changes include nuclear condensation, aggregation of chromatin at the nuclear margins, cell shrinkage, blebbing of the plasma membrane, and cleavage of DNA into fragments corresponding to nucleosomes (so-called DNA laddering), accumulation of reactive oxygen species (ROS), release of cytochrome *c* from mitochondria, and activation of a number of hydrolytic enzymes such as serine and cysteine proteases and DNase, events that are all commonly observed during animal apoptosis.

In spite of the observations of these classic cellular morphological and biochemical changes described for animal apoptosis in plant PCD systems, sequenced plant genomes revealed the absence of plant homologues to several key regulators of animal PCD, including canonical caspases and Bcl-2-related proteins. Nevertheless, caspase-like protease (CLP) activities are transiently activated in plants synchronized to undergo plant PCD induced by biotic or abiotic stress [13–16]. Moreover, it was shown that ectopic expression of the pro-apoptotic protein Bax can induce cell death with apoptotic features and conversely, overexpression of metazoan anti-apoptotic proteins such as IAP (Inhibitor of Apoptosis) and Bcl-X_L_ proteins can suppress some types of plant PCD [17–20]. From genetic studies, many genes have been identified to affect PCD signaling in plants as exemplified by so-called “disease lesion mimic” mutants in various plant species including Arabidopsis and maize [21,22]. Together, these observations strongly suggest that plant should possess a programmed mechanism similar to but distinct from animal PCD. In this scenario, PCD in plants may be controlled via a distinct set of regulators that performs similar functions to animal PCD regulators. Therefore, understanding of the mechanisms and pathways that mediate PCD in plants will benefit our knowledge to improve plant tolerance to biotic and abiotic stress by manipulating cell death mechanism under these conditions.

PCD has been mainly described for multicellular organisms including plants and animals but individual cell death via programmed mechanism against stress stimuli is believed to be important for the maintenance of the population for unicellular eukaryotes such as fungi [23]. Similar to plants, yeasts lack much of the molecular machinery that is responsible for apoptosis in metazoans [24–26]. However, it was shown that Bax-mediated cell death in the budding yeast *Saccharomyces cerevisiae* is accompanied by typical features of apoptosis, such as membrane blebbing, chromatin condensation and DNA cleavage, cytochrome c release from mitochondria, and externalization of phosphatidylserine onto the surface of the cytoplasmic membrane [27,28]. The co-expression of BCL-X_L_ or mammalian Bax Inhibitor-1 (BI-1) prevents these effects and cell death activation by Bax [27,29,30]. Cell death with apoptosis-like features has also been reported in yeast after treatment with acetic acid, UV-irradiation, glutathione-depleting chemicals and H_2_O_2_ [31–33]. Additionally, it has been revealed that yeasts possess several orthologues for mammalian cell death regulators or mediators, *i.e.* Omi/HrtA2, AIF, and EndoG [34–36], suggesting that the basic machinery of apoptosis is indeed present and functional also in unicellular eukaryotes. Therefore, it is very important to compare the differences in the control of PCD between diverse organisms from an evolutionary aspect. Understanding of how PCD mechanisms may have evolved will contribute to our understanding of the key steps that are fundamental to orchestrating PCD and the various mechanisms that have been elaborated during evolution for its regulation in diverse cellular context.

The ER is a highly evolved organelle that has specific mechanisms to ensure nascent protein synthesis, oxidative protein-folding and post-translational modification such as *N*-glycosylation, as well as its own homeostasis in the lumen of ER. When these mechanisms are impaired, cellular function can become compromised and leads to a disturbed cellular condition referred to as “ER stress” [37,38]. Adaptation to recovery from ER stress is mediated by defense mechanisms through the so-called unfolded protein response (UPR) or ER stress response that integrates signals from pathways that transmit information about protein-folding status in the ER lumen to the cytosol and nucleus to increase protein-folding capacity via conserved transcriptional regulation [38]. However, cells will undergo apoptosis if these mechanisms for stress adaptation are insufficient to relieve the ER stress induced by extensive or prolonged input from the UPR. Recent emerging evidence demonstrated the existence of ER-dependent apoptosis pathway that is initiated when ER homeostasis is heavily impaired due to ER stress [39]. It is evident from a number of studies that plants possess such defense mechanisms to cope with ER stress [40–46], although the UPR in plants still remains unclear at the molecular and biochemical levels. Like mammalian BI-1, plant BI-1 is also reported to be an ER-resident protein [47–50], it has thus been suspected that BI-1 function may be associated with ER-related cellular activity such as homeostasis control by the UPR or ER stress response [51].

In this review, we describe current knowledge on role of BI-1, an evolutionary conserved ER resident protein that suppresses cell death induced by biotic and abiotic stresses. Here, we also discuss the recent exciting findings on the functional importance of plant and animal BI-1 in the ER-dependent cell death pathway. Finally, the possible scenario of how plant BI-1 could suppress a variety of stress-induced cell death in plants is explored.

## 2. Bax Inhibitor-1 (BI-1): History and Functional Implication in PCD

The yeast system has been used as an excellent model system to carry out screens for cDNAs which encode proteins that either enhance or inhibit apoptotic phenotypes. Bax inhibitor-1 was originally isolated from a human cDNA library because of its ability to block cell death induced by the ectopic expression of the mouse Bax gene in yeast [30]. The predicted BI-1 protein sequence shows high identity (90%) to a testis enhanced gene transcript from a cDNA library of adult rat testis [52]. Overexpression of human BI-1 resulted in protection against apoptosis induced by certain types of cell death stimuli such as etoposide, staurosporine, and growth factor depletion but not Fas (CD95). Conversely, BI-1 antisense induced apoptosis in cancer cell lines, suggesting a new type of regulator of cell death pathways controlled by Bcl-2 and Bax [30]. Interestingly BI-1 cannot physically interact with Bax, indicating that BI-1 does not inhibit Bax activity directly and may act downstream of the site of Bax action. However, a direct physical interaction between BI-1 and Bcl-2 has been observed by *in vivo* cross-linking and co-immunoprecipitation experiments [30]. BI-1 might enhance the anti-apoptotic action of Bcl-2, although the significance of the interaction of BI-1 with Bcl-2 remains to be clarified. Intracellular localization of mammalian BI-1 was determined by subcellular fractionation, immunostaining and GFP fusion approaches, indicating that BI-1 proteins associates with intracellular membranes, especially showing a pattern typical of ER and its continuity with the nuclear envelope. BI-1 transcript was detected widely in most organs and tissues examined and remarkably abundant in cancer cell lines [30], suggesting that animal BI-1 may have an anti-apoptotic function.

Plant BI-1 homologues were cloned from *Arabidopsis* and rice, and their ability to rescue Bax-induced lethality in yeast confirmed [53]. Subsequently BI-1 related genes from several plant species have also been characterized to various extent [54,55] (see Table 1). Like mammalian BI-1, plant BI-1 genes also expressed in diverse tissue types and their expression levels are enhanced during aging (senescence) and under stress conditions [47,48,50,56–59]. Importantly, Kawai-Yamada *et al.* [47] reported that *Arabidopsis* BI-1 (AtBI-1) fused to GFP is also observed at the ER and the nuclear envelop, and its overexpression can suppress Bax-induced lethality in *Arabidopsis* plants, providing direct molecular evidence that plant BI-1 can be functional to inhibit mammalian Bax action *in planta*. Interestingly, elevated intracellular ROS levels by Bax expression was not abrogated in AtBI-1 overexpressing *Arabidopsis* plants, although cell death was strongly suppressed. Kawai-Yamada and co-workers also showed that hydrogen peroxide-induced cell death of tobacco BY-2 cells can be protected by AtBI-1 overexpression, although relative ROS level was not significantly modulated [59]. On the other hand, several plant ROS scavenging enzymes such as Fe-SOD, GST, APX and PHGPx were isolated as “Bax Inhibitors” from a cDNA library screen using the yeast-Bax survival screen [60–63]. This implies that ROS accumulation is an important step to mediate Bax-dependent cell death pathway in yeast. ROS generated by stresses can act as mediators that function at the early stage in signal transduction, stress response and PCD [11,12,24,25]. Although overexpression of plant BI-1 from various plant species was shown to inhibit fungal pathogen-, fungal elicitor-, hydrogen peroxide-, salicylic acid-, heat- and cold-induced cell death, in some cases stress-induced ROS was not significantly changed in BI-1 overexpressing cells [59]. Thus, plant BI-1 may function downstream from the early steps of ROS-dependent cell death pathway.

It has been reported that transgenic plants expressing animal or viral regulators of apoptosis (Bcl-2, Bcl-X_L_, Bax, CED-9, Op-IAP, or p35) display specific phenotypes (promotion or suppression of cell death activation) during pathogen challenge depending on the nature of the transgene [17–20,64,65]. Involvement of plant BI-1 in the hypersensitive response (HR), a well-characterized form of plant PCD, has been indicated in some cases [56,58,66,67]. HR-PCD appears to play different roles depending on the lifestyle of pathogens. In the interaction of plants with biotrophic pathogens, HR-PCD is a central part of the resistance mechanism to restrict pathogen growth and spread. In contrast, during the interaction of plants with necrotrophic pathogens, cell death apparently provides a benefit to their successful infection and growth on the host [68]. Thus, inducing intrinsic PCD in a host plant cell may be part of the invasion strategy for necrotrophs but not for biotrophs. Using plant-fungal pathogen interaction systems, Hückelhoven and his co-workers demonstrated that overexpression of plant BI-1 confers reduced or enhanced succeptibility to distinct fungal pathogens in barley and carrot plants [49,50,66,67,69,70]. In addition, overexpression of barley BI-1 can confer resistance to the root endophytic fungus that requires host cell death for proliferation in the differentiation zone of barley roots [71], supporting the idea that plant BI-1 has anti-PCD function in plants in response to fungal pathogens with different lifestyles (see Table 1). How plant BI-1 proteins protect cells against bacterial and viral pathogens remains to be determined.

Using reverse genetic approach, recent studies demonstrated that AtBI-1 plays a role in suppressing biotic and abiotic types of cell death [72]. In fact, two *AtBI-1* mutants, *atbi1-1* and *atbi1-2* (a gene knock-out) exhibit accelerated progression of cell death upon infiltration of leaf tissues with a PCD-inducing fungal toxin fumonisin B1 (FB1) or against heat shock (see Table 1). Under these conditions, expression of AtBI1 mRNA was strongly up-regulated in wild-type leaves prior to the activation of cell death, suggesting that increase of AtBI1 expression is important for basal inhibition of cell death induction [72]. However, these two mutants displayed no obvious phenotype under normal growth conditions. Similar to Arabidopsis BI-1 mutants, BI-1-deficient mice also do not exhibit any histological abnormalities in tissues and organs examined [51], suggesting that BI-1 is dispensable for physiological regulation of developmental PCD in plants and animals. Taken together, these findings indicate that BI-1 is a conserved cell death suppressor for stress-induced PCD pathways in plants and animals.

## 3. Structure, Functional Domain and Plausible Biochemical Activity of BI-1 Proteins

**Figure 1.**
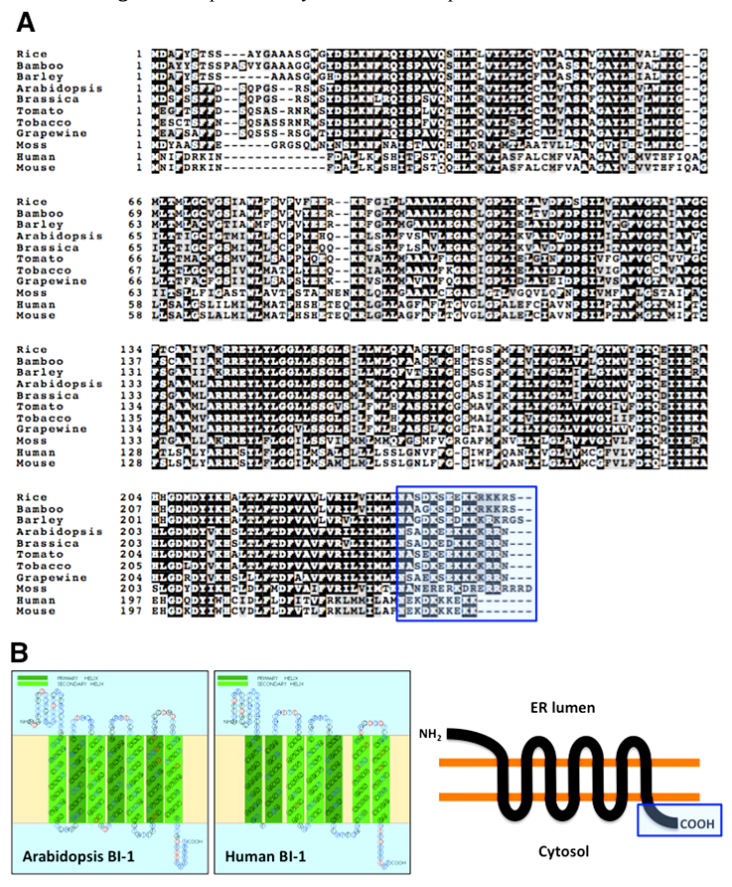
Sequence analysis of BI-1 from plants and mammals. (A) Alignment of the predicted amino acid sequences of BI-1 from plants and mammals. Conserved C-terminal domains for each BI-1 is highlighted. Accession numbers for each BI-1 are bellows: Rice (AB025926), Bamboo (DQ227647), Barley (AJ290421), Arabidopsis (AY091134), Brassica (AF390555), Tomato (AY380778), Tobacco (AF390556), Grapewine (AM487249), Moss (A9SK21), Human (NP003208), Mouse (NP080945). (B) Predicted membrane topology of Arabidopsis and human BI-1 proteins.

BI-1 proteins are evolutionary conserved small proteins (25 to 27 kDa) that predominantly localize to the ER membrane. As shown Figure 1A, BI-1 proteins from plants and mammals are very homologous. They show very high similarity in their entire amino acid sequences between plants and animals. Based on structural model of BI-1 proteins derived from the SOSUI program, which can predict the most favored membrane topology (http://bp.nuap.nagoya-u.ac.jp/sosui/sosuiframe0.html) [73], *Arabidopsis* and human BI-1 proteins are likely to contain seven transmembrane alpha-helices followed by a C-terminal domain that is exposed on the cytosolic side of the membrane (Figure 1B). This prediction for both plant and animal BI-1 proteins is supported by results from detergent partitioning studies [30,48]. *N*-termini of BI-I proteins are predicted to reside in the lumen side of ER. Of note, BI-1 proteins do not contain any of the well-characterized motifs for post-translational modification. However, BI-1 proteins contain highly hydrophilic *C*-terminal domains consisting of short clusters of charged amino acids, a feature that is well conserved in BI-1 proteins from plants and animals (Figure 1B). From secondary structure analyses, this region is predicted to form a coiled-coil structure that might be important for regulating the BI-1 activity as a cell death suppressor. In fact, deletion or modification of their *C*-terminal sequences (*i.e.* losing or reducing the possibility of coiled-coil structure forming properties) abrogated the cytoprotective ability of BI-1 to suppress BAX-induced cell death in yeast cells [59,74]. In addition, importance of an intact *C*-terminal domain for AtBI-1 was demonstrated by genetic analysis indicating that *atbi1-1* mutants, expressing a mutant form of AtBI-1 protein with a *C*-terminal missense mutation, show increased sensitivity to fungal toxin-induced and heat shock-induced PCD in *Arabidopsis* [72]. *atbi1-1* mutants also show increased sensitivity to a Ca^2+^-pump inhibitor (cyclopirazonic acid; CPA) or upon ion stress under low Ca^2+^ and high Mn^2+^ conditions that can induce cell death in plants [75].

The predicted membrane topology of BI-1 suggests the possibility that this protein may act as a transporter of ions or other types of molecules across the ER membrane. In animal cells, BI-1 was shown to selectively protect cells against apoptosis induced by ER stress inducers such as the *N*-glycosylation inhibitor tunicamycin and the calcium pump inhibitor thapsigargin [51]. Apparently overexpression of BI-1 results in the reduction of free Ca^2+^ concentration in the ER [51,76]. Kim *et al.* [77] found that the *C*-terminal sequence of BI-1 (EKDKKKEKK) is very similar to the pH-sensing domain of mechanosensitive channel (MSCL) that mediates gating of this ion channel in a pH-dependent manner [78], thereby leading them to hypothesize that BI-1 may function as a pH-sensitive regulator of calcium channel activity on the ER membrane. Their biochemical analysis indicated that weakly acidic conditions (e.g. pH 5.4) triggered more extensive Ca^2+^ release from BI-1 overexpressing ER but not from C-terminal deleted BI-1 overexpressing cells [77]. Surprisingly, BI-1 overexpression triggered apoptosis due to extensive Bax recruitment to the mitochondria and cytochrome c release from mitochondria under acidic conditions, prompting a novel proposal that BI-1 can possess “cell death-promoting” activity that can become apparent under conditions of low intracellular pH. Using a BI-1-reconstituted liposome system, human recombinant BI-1 was shown to have Ca^2+^/H^+^ antiporter-like activity *in vitro* [79]. In this system, BI-1 mediates an efflux of Ca^2+^ from Ca^2+^ loaded liposomes with concomitant influx of H^+^ into the liposomes depending on the acidic pH (5.0–6.5), while Ca^2+^/H^+^ antiporter-like activity of BI-1 was not detected under more physiological pH conditions. Thus, BI-1 activity may be controlled by alterations in cytosolic pH during apoptosis. Overall, mammalian BI-1 may have two separate functional domains for its channel function as well as a protein-protein interaction domain that can modulate the biochemical activity of its associated protein(s) in mammalian cells under normal and stress conditions (see Section 4).

## 4. Role of Mammalian BI-1 in the ER-dependent PCD Pathway

In mammalian cells, accumulation of unfolded and misfolded proteins in the lumen of the ER can be triggered by a diverse array of cellular stresses that are induced by multiple stimuli and pathological conditions. The consequence of triggering the ER stress response in mammalian cells can be grouped into three types of signaling pathways mediated by ER-resident membrane proteins: adaptation, alarm and apoptosis [37–39]. The tight regulation of the adaptation process is a fundamental mechanism to reestablish homeostasis and normal ER functions. This mechanism is mediated by conserved activation of transcriptional programs that induce expression of genes capable of enhancing the protein folding capacity in the ER and genes for ER-assisted degradation [38]. A recent study by Lisbona *et al.* [80] demonstrated that mammalian BI-1 plays a role in early adaptive response against ER stress through its direct interaction with the ER stress sensor protein IRE1-α, which activates its direct downstream target gene X-box-binding protein-1 (XBP-1) and followed by up-regulation of genes that encode ER-resident chaperones. The formation of a protein complex between BI-1 and IRE1-α was shown to be mediated through the *C*-terminal domain of BI-1 at the ER membrane. This regulation did not affect the IRE1-α/XBP-1 independent ER stress response pathways mediated by ATF6 or PERK. The IRE1-α/XBP-1 pathway was hyperactive and insensitive in BI-1 knock-out cells and BI-1 overexpressing cells, respectively, in response to ER stress-inducing drugs [80]. Importantly, the results of Lisbona *et al.* [80] indicated that the ER stress response can be suppressed by BI-1 under mild ER stress conditions but not under severe stress conditions, suggesting that BI-1 functions is required during the early stage of adaptive ER stress response to prevent activation of PCD.

Another recent study revealed the complex mechanisms controlling BI-1 function in mammalian cells. In fact, BI-1 can modulate ER stress-induced ROS accumulation through regulation of cytochrome P450 2E1 (P450 2E1) activity via indirect binding to an NADPH-dependent cytochrome c P450 oxidoreductase (NPR), whose interaction is mediated through the *C*-terminal domain of BI-1 [81]. Uncoupling of electron flow between cytochrome P450 2E1 and NPR is known to be a major source of ROS on the ER membrane, thus modulation of electron flow from NPR to P450 2E1 by BI-1 can result in a reduction of ROS production that potentially attenuates unfolded and misfolded proteins accumulation in the ER, thereby reducing cell death. This finding may be important to note that ER stress-associated ROS production initiated from the ER could result in the dysfunction of mitochondria, especially inhibiting its respiration activity, thereby triggering the activation of mitochondria-dependent apoptosis pathway.

## 5. Role of Plant BI-1 in ER-dependent PCD Pathway

The existence of ER stress-mediated apoptosis pathway and functional implication of mammalian BI-1 linking to the protection of cells from ER stress-induced PCD has been established [51,80–84]. In contrast, these aspects have not been well documented in any plant systems. We recently demonstrated that in *Arabidopsis* seedlings, strong PCD phenotypes such as nuclear condensation, DNA laddering and H_2_O_2_ production were observed in roots that have been challenged with various drugs known to induce ER stress such as the *N*-linked glycosylation inhibitor tunicamycin (TM), CPA, and the proline analogue l-azetidine-2-carboxylic acid (AZC) [85]. Since *AtBI-1* expression is induced transcriptionally through an UPR pathway prior to cell death induction by these ER-stress inducers [43,85], BI-1 level could be an important determinant for plant survival during ER stress in *Arabidopsis* plants. In fact, ER stress-mediated PCD can be manipulated by the disruption of *AtBI-1* or overexpressing AtBI-1 proteins, resulting in accelerated or attenuated PCD, respectively [85]. Expression of UPR genes, however, are unaffected by changes in AtBI1 levels, thereby suggesting a scenario in which AtBI1 does not control the UPR directly, but instead may act as a cell death suppressor working in parallel to the UPR after the initial activation of ER stress.

In animal apoptosis, mitochondria-dependent pathway plays an important role for the execution of cell death, including the release of cytochrome *c* and loss of mitochondrial membrane potential, changes in electron transport as well as participation of pro-and anti-apoptotic Bcl-2 family proteins that may localize to the mitochondrial membrane [7,24]. Kawai-Yamada and co-workers also identified a yeast mutant Δcox16, which is deficient in a subunit of the cytochrome *c* oxidase (COX) complex, that AtBI-1 fails to rescue from Bax-induced lethality [86]. Similar growth phenotype was also observed in other COX-deficient strains (Δcox6, Δcox7, Δcox11, Δcox15, Δcox18), indicating that functional mitochondrial respiration activity including ATP synthesis is indispensable for BI-1-mediated protection from Bax-induced cell death of yeast. Interestingly, mouse Bcl-2 was shown to rescue all yeast COX mutant strains tested from Bax-induced lethality [86]. This could be explained by the fact that Bcl-2 inhibits Bax action at the mitochondria directly while BI-1 inhibits Bax action indirectly, providing further evidence that BI-1 and Bcl-2 inhibit Bax-mediated yeast cell death through different mechanisms. Thus, the ability of an ER-localized BI-1 to suppress pro-death signals being generated from the mitochondria (*e.g.* via Bax expression) implies the existence of secondary mediators, perhaps Ca^2+^ and ROS, that are involved in cross-talk between the ER and mitochondrial cell death pathways. A recent mammalian BI-1 study revealed a hierarchy of functional interactions of BI-1 with Bcl-2 and Bax proteins showing that BI-1 and Bcl-2 control apoptosis downstream of or parallel to Bax action through regulating Ca^2+^ homeostasis in the ER [84]. Therefore, Bax might cause dysfunction of ER homeostasis through facilitating Ca^2+^ release from the ER. In plants there are no obvious homologues to Bcl-2 subfamily member, thus plant BI-1 may play a major role in regulating Ca^2+^ homeostasis in the ER during the onset of Bax-induced PCD.

Ca^2+^ is a universal intracellular messenger that can control a broad range of cellular processes in plants but only a small number of calcium related genes have been directly implicated in plant cell death control [87]. Kawai-Yamada and co-workers recently reported that *Arabidopsis* calmodulin-7 (AtCaM7), a calcium-binding protein that facilitate Ca^2+^-dependent cellular response by modulating biochemical activities of their target proteins, can interact with AtBI-1 in yeast and tobacco cells via its C-terminal domain [75]. Functional importance of CaM-AtBI-1 interaction in PCD control remains to be determined. However, it was evident that overexpression of AtBI-1 can modulate cell death of tobacco cells induced by a ER-resident calcium pump inhibitor cyclopiazonic acid (CPA) that cause ER stress in animal cells [75]. Using several yeast knock-out mutants, Kawai-Yamada and co-workers also found that AtBI-1 fails to rescue Bax-activated yeast cell death in yeast mutants lacking Ca^2+^-ATPase (Ca^2+^ pump) located on ER membrane but not other Ca^2+^ pumps located on the plasma membrane or vacuole [75]. These observations suggest a possible connection between AtBI1 and calcium homeostasis specifically at the ER.

The importance of sphingolipid fatty acid metabolism in plant, yeast, and animal cell death pathways has been suggested [88–93]. Sphingolipids consist of a diverse group of lipids that contain a relatively large hydrophobic moiety, known as ceramides, that include a sphingoid or long-chain base amide linked to a fatty acid. Sphingolipids act as second messengers to regulate stress response, cell proliferation and apoptosis in mammals and the balance between the bioactive sphingolipid ceramide and its phosphorylated derivative has been proposed to potentiate PCD in eukaryotes [88]. AtBI-1 has been implicated in the regulation of PCD associated with alterations in sphingolipid metabolism caused by a mycototoxin fumonisin B1 that triggers PCD in plants [72]. It was also reported that AtBI-1 may function as a cell death suppressor through direct interaction with an AtCb5-FAH1 protein complex at the ER membrane, which is involved in 2-hydroxylation of sphingolipids [94]. From functional screening using the split-ubiquitin yeast two-hybrid system (suY2H system) with *Arabidopsis* cDNAs*,* the highly conserved electron transport protein cytochrome *b*_5_ (AtCb5) was identified as a possible protein interaction partner of AtBI-1. In the *Arabidopsis* genome, five genes encoding Cb5 isoforms (AtCb5A-E) and one Cb5-like gene (AtCb5LP) have been annotated. Among them, all AtCb5 isoforms (AtCb5A-E) were shown to interact with AtBI-1 in yeast cells. Yeast has several genes encoding proteins homologous to AtCb5 and/or related to fatty acid metabolism (Cb5, fatty acid hydroxylase (FAH1), C-22 sterol desaturase, cytochrome *b5* reductase, NADH-cytochrome *b5* reductase). Of these, only FAH1 was shown to be functionally required for AtBI-1-dependent protection against Bax-induced lethality and their direct interaction was shown in the suY2H system [94]. Interestingly, yeast FAH1 (ScFAH1) has two functional domains: one is a Cb5-like region at the *N*-terminus and the other resembles a fatty acid 2-hydroxylase domain [95]. *Arabidopsis* has two homologous genes to ScFAH1, so-called AtFAH1 and AtFAH2, that contain one fatty acid 2-hydroxylase domain only. Since Cb5-like domain is essential for the catalytic activity of human fatty acid 2-hydroxylase, AtFAH and AtCb5-B may form a protein complex that is active in lipid modifications in *Arabidopsis* plants. In fact, physical interaction between AtCb5-B and AtBI-1 at the ER membrane was confirmed by a bimolecular fluorescence complementation (BiFC) assay and fluorescence resonance energy transfer (FRET) analysis via transient expression assays in onion epidermal cells [94]. Thus, three *Arabidopsis* proteins (AtBI-1, AtCb5-B and AtFAH) can form a large protein complex and biochemical activities of AtBI1 and AtCb5-AtFAH may be tightly associated. The analysis of total 2-hydroxy fatty acids composition also showed that overexpression of AtBI-1 increases the relative level of 2-hydroxyl fatty acids (2-HFA) with long lipid side chains including C22 h:0, C24 h:0, C24 h:1, C26 h:0 and C26 h:1, although the level of all 2-HFA in *atbi1-1* mutant and *AtBI1* knock-down lines were similar to that in wild-type *Arabidopsis* plants [94]. This observation suggests that there may be additional proteins in *Arabidopsis* that is functionally redundant to AtBI-1 in the wild-type background which can work together with AtCb5-B and AtFAH, and the activity of AtBI-1 only becomes apparent when it is more highly expressed. Thus, it is hypothesized that AtBI-1 might be able to promote AtCb5-AtFAH activity in wild-type plants under stress conditions when BI-1 protein levels are up-regulated. Quantitative analyses of 2-HFAs in wild-type, *atbi-1* mutants, or AtBI-1 overexpressors under different stress situations will be interesting to test this hypothesis and address the question of how AtBI-1 may modulate the sphingolipid-associated PCD pathway in plants.

## 6. Working Model of Plant BI-1 in PCD Induced by Biotic and Abiotic Stresses

**Figure 2.**
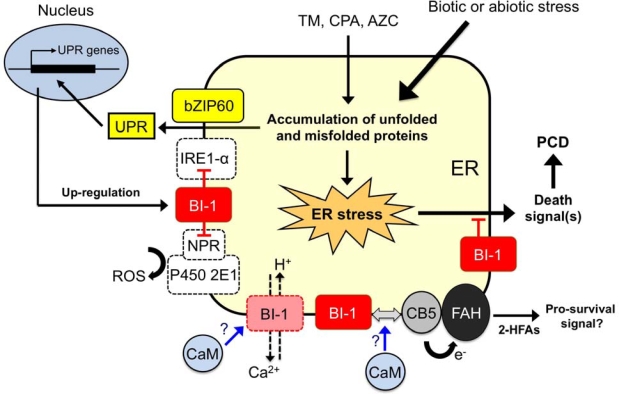
Working model of plant BI-1 in the control of PCD induced by ER stress, biotic or abiotic stress. See text for explanation.

PCD in plants is generally accompanied by changes in cellular redox status and cytosolic Ca^2+^ levels upon challenge with cell death stimuli including ER stress. At this point, the following model can be suggested for the role of BI-1 in controlling plant PCD (Figure 2). Under weak or mild ER stress conditions induced by less than sub-lethal doses either of TM, CPA or AZC, ER stress response is consequently activated to express a set of UPR genes such as BIP2 and CRT1 to restore ER homeostasis [85]. Plant BI-1 may also be induced transcriptionally through a similar pathway, perhaps by one of the ER-resident bZIP transcription factors AtZIP60 [46]. This up-regulation of BI-1 should increase the amount of BI-1 on the ER membrane and support its activity as a survival factor involved in an anti-PCD pathway. According to the recent findings from Kawai-Yamada’s lab as well as by analogy to findings with animal BI-1, binding of calmodulin with plant BI-1 may alter its biochemical activity through its C-terminal region, *i.e.* Ca^2+^/H^+^-like channel activity or in association with the AtCb5-AtFAH protein complex [75,77,79,94]. At this point, it remains unclear whether calmodulin modulates these possible BI-1 activities either positively or negatively and the cellular context under which the calmodulin interaction with BI-1 may be operational. In one scenario, under stress conditions calmodulin may facilitate disassociation of BI-1-AtCb5-AtFAH complex that can lead to suppressed 2-HFAs generation. As the steady-state levels of 2-HFAs are increased in AtBI-1 overexpressing cells, this up-regulation may play a role in stress adaptive response downstream of ER stress response induced by oxidative stress or inhibiting Ca^2+^-pump activity on the ER membrane [94]. Since loss-of-function of AtBI-1 has been found to result in increased ROS accumulation under ER stress [85], ER-associated ROS generation that is associated with plant homologues encoding NPR and P450 2E1 may also be involved in an ER stress-mediated cell death pathway in plants [81]. In fact, three homologous genes encoding putative NPR and many genes encoding P450 2E1-like protein have been annotated in the *Arabidopsis* genome. In addition, direct association between BI-1 and IRE1-α may control IRE1-α-dependent UPR pathway, which is required for suppression of intense ER stress response that might trigger PCD in plants [80]. As plant IRE1 homologues have been identified and characterized [40,44], it should be possible to test this hypothesis. However, increase in BI-1 expression as well as the ER stress response will not be sufficient to relieve persistent or intense ER stress conditions, ultimately leading to cell death as a protective step to remove damaged cells from the organism [80,85]. Overall, AtBI1 is a critical survival factor for suppression of PCD induced by mild ER stress and allow the UPR sufficient time to re-establish proper homeostasis in the cell (Figure 2).

With respect to the postulated role of the ER in plant PCD, it should be noted that *Arabidopsis* DAD1 (defender against death-1), an ER-resident protein that mediates *N*-glycosylation of nascent proteins, has been found to play a role in suppressing PCD induced by salt stress or UV-C irradiation [96,97]. Furthermore, an intact and responsive protein secretion pathway with up-regulation of secretion-related genes such as Bip, PDI, CRT, and DAD1 was shown to be essential for the induction of systemic acquired resistance against biotic stresses during challenge by bacterial pathogens in *Arabidopsis* [98]. In addition, we reported that application of chemical chaperones, small molecules that were able to relieve ER stress by mimicking action of protein chaperones in animal cells [99], were able to suppress induction of the UPR and plant cell death in whole *Arabidopsis* seedlings even under extensive and prolonged ER stress conditions [85]. Thus, multiple types of abiotic and biotic stresses may potentially trigger similar perturbations in ER homeostasis followed by the ER stress response and cell death activation (see Figure 2). In this regard, the observed role of plant BI-1 as a highly conserved cell death suppressor in eukaryotes under diverse stress conditions may be explained (see Table 1 and Figure 2). To further examine this hypothesis, it will be interesting to determine whether different unrelated biotic and abiotic stresses can trigger similar perturbation of ER homeostasis followed by the ER stress response and BI-1 potentiated cell death induction.

## 7. Conclusions

In this article, we provide an update on the role of BI-1 in the control of PCD under stress conditions. At present, BI-1 is a candidate for an evolutionary conserved, ER-resident protein that represents an ancient cell death regulator that potentially regulates PCD in all eukaryotes. The ER has emerged recently as a conserved site where cell death signals in the forms of ROS and Ca^2+^ can be generated. In plants, lipid modification and generation of 2-hydroxyl fatty acids on the ER membrane seem likely to be important steps to modulate cell death activation. Ultimately, further studies will be required to sort out the complex interplay between these cell death signals and the variety of regulators that are likely to control PCD. In this regard, elucidating the mode of action of BI-1 through structure/function studies will provide important clues to the evolution of PCD in eukaryotes. This knowledge may make possible future biomedical and agricultural applications for relieving various types of cell death related diseases that may be activated through dysfunctions of the ER stress signaling pathway. Furthermore, understanding of the mechanisms and pathways that mediate PCD in plants should aid our effort to improve plant tolerance to biotic and abiotic stresses by genetic engineering and/or chemical biological approaches that manipulate plant cell death processes under these conditions.

## Figures and Tables

**Table 1.** Stress-induced cell death controlled by BI-1 in plants.

**Species/organ, cell culture**	**BI-1 expression**	**Cell death system (IC or SC)**	**Response/Phenotype**	**References**

*A. thaliana*/seedlings	OE	Mouse BAX_IC	Decreased sensitivity	[47]
*A. thaliana* /seedlings	KO	Heat shock_IC	Increased sensitivity	[72]
*A. thaliana* /mature plants	KO	Heat shock_IC	Increased sensitivity	[72]
*A. thaliana* /mature leaves	KO	Fungal toxin (FB1)_IC	Increased sensitivity	[72]
*A. thaliana* /root cells	OE	Tunicamycin_IC	Decreased sensitivity	[85]
*A. thaliana* /root cells	KO	Tunicamycin_IC	Increased sensitivity	[85]
*D. carota*/whole plants	OE	Fungal pathogen^a^_IC	Decreased susceptibility	[69]
*D. carota*/whole plants	OE	Fungal pathogen^b^_IC	Decreased susceptibility	[69]
*H. vulgare*/leaf epidermal cells	OE	Fungal pathogen^c^_SC	Increased susceptibility	[50]
*H. vulgare*/seedlings	OE	Fungal pathogen^c^_SC	Increased susceptibility	[66]
*H. vulgare*/leaf epidermal cells	OE	Fungal pathogen^d^_IC	Decreased susceptibility	[67]
*H. vulgare*/whole plants	OE	Fungal pathogen^e^_IC	Increased resistance	[70]
*H. vulgare*/root cells	OE	Fungal symbiont^f^_IC	Decreased colonization	[71]
*N. tabacum*/BY-2 cells	KD	Sucrose starvation_IC	Accelerated cell death	[57]
*N. tabacum*/BY-2 cells	OE	Salicylic acid (SA)_IC	Decreased sensitivity	[59]
*N. tabacum*/BY-2 cells	OE	Hydrogen peroxide_IC	Decreased sensitivity	[59]
*N. tabacum*/BY-2 cells	OE	Cyclopiazonic acid_IC	Decreased sensitivity	[75]
*N. tabacum*/leaf discs	OE	Heat shock_IC	Decreased sensitivity	[74]
*N. tabacum*/leaf discs	OE	Cold shock_IC	Decreased sensitivity	[74]
*O.sativa*/suspension culture	OE	Fungal elicitor_IC	Decreased sensitivity	[58]
*O.sativa*/suspension culture	OE	Salicylic acid (SA)_IC	Decreased sensitivity	[58]

OE: overexpression; KD: knock-down; KO: knock-out; IC: inducing cell death; SC: suppressing cell death;

^a^ *Botrytis cinerea* (necrotroph);

^b^ *Thielaviopsis brassicicola* (necrotroph*)*;

^c^ *Blumeria graminis* (biotroph*)*;

^d^ *Fusarium graminearum* (necrotroph);

^e^ *Phakopsora pachyrhizi* (basidiomycete);

^f^ *Piriformosprora indica* (root endophyte).
